# Impact of sars-cov-2 interventions on dengue transmission

**DOI:** 10.1371/journal.pntd.0008719

**Published:** 2020-10-29

**Authors:** Jue Tao Lim, Borame Sue Lee Dickens, Lawrence Zheng Xiong Chew, Esther Li Wen Choo, Joel Ruihan Koo, Joel Aik, Lee Ching Ng, Alex R. Cook

**Affiliations:** 1 Saw Swee Hock School of Public Health, National University of Singapore and National University Health System, Singapore; 2 Department of Geography, Faculty of Arts and Social Sciences, National University of Singapore, Singapore; 3 Department of Biological Sciences, Faculty of Science, National University of Singapore, Singapore; 4 Environmental Health Institute, National Environmental Agency, Singapore; Georgia Southern University Jiann-Ping Hsu College of Public Health, UNITED STATES

## Abstract

An estimated 105 million dengue infections occur per year across 120 countries, where traditional vector control is the primary control strategy to reduce contact between mosquito vectors and people. The ongoing sars-cov-2 pandemic has resulted in dramatic reductions in human mobility due to social distancing measures; the effects on vector-borne illnesses are not known. Here we examine the pre and post differences of dengue case counts in Malaysia, Singapore and Thailand, and estimate the effects of social distancing as a treatment effect whilst adjusting for temporal confounders. We found that social distancing is expected to lead to 4.32 additional cases per 100,000 individuals in Thailand per month, which equates to 170 more cases per month in the Bangkok province (95% CI: 100–242) and 2008 cases in the country as a whole (95% CI: 1170–2846). Social distancing policy estimates for Thailand were also found to be robust to model misspecification, and variable addition and omission. Conversely, no significant impact on dengue transmission was found in Singapore or Malaysia. Across country disparities in social distancing policy effects on reported dengue cases are reasoned to be driven by differences in workplace-residence structure, with an increase in transmission risk of arboviruses from social distancing primarily through heightened exposure to vectors in elevated time spent at residences, demonstrating the need to understand the effects of location on dengue transmission risk under novel population mixing conditions such as those under social distancing policies.

## Author summary

Significant healthcare burdens arise from dengue and traditional vector control is the primary control strategy to reduce dengue transmission potential. The ongoing sars-cov-2 pandemic has resulted in dramatic reductions in human mobility due to social distancing measures; the effects on vector-borne illnesses are not known. This paper examines the pre and post differences of dengue case counts in Malaysia, Singapore and Thailand, and estimate the effects of social distancing as a treatment effect whilst accounting for temporal confounders. We found that social distancing is expected to lead to an increase in the number of cases in Thailand, with the largest increase arising from Bangkok province. Social distancing policy estimates for Thailand were also found to be robust in sensitivity analysis. Conversely, no significant impact on dengue transmission was found in Singapore or Malaysia. Across country disparities in social distancing policy effects on reported dengue cases are reasoned to be driven by differences in workplace-residence structure, with an increase in transmission risk of arboviruses from social distancing primarily through heightened exposure to vectors in elevated time spent at residences, demonstrating the need to understand the effects of location on dengue transmission risk under novel population mixing conditions such as those under social distancing policies.

## Introduction

An estimated 105 million dengue infections occur per year, with case counts reported in over 120 countries [1, 2]. Southeast Asia and the Western Pacific region is estimated to bear 75% of the global burden of dengue [3]. The Southeast Asia region comprises of 11 countries with a total population of 1.97 billion [4] and the Western Pacific Region comprises of 37 countries with a total population of 1.9 billion [5, 6] i.e. more than North or South America. Around 2.9 million dengue cases are reported annually in the region, with high incidence year-round, resulting in over 5500 dengue-related deaths on average [7]. The dengue virus is transmitted primarily by the *Aedes aegypti* and *Ae*. *albopictus* vectors. The tropical-subtropical climate of Southeast Asia is suitable [8] for the vector and in conjunction with increasing human population connectivity, population density, global warming and urbanisation within the region, creates an ideal environment for dengue transmission to occur [9–11]. Urban centers in Southeast Asia also often report the highest number of dengue case counts, which are postulated to seed outbreaks in other localities [9] and increases in temperatures due to climate change allow for optimal vector breeding conditions, shorten the incubation period of the dengue virus and increase the overall risk of dengue disease within the region [12].

Four serotypes of the dengue virus exist and predominate in different localities [13]. The active circulation of all four serotypes in Southeast Asia, where dengue is hyper-endemic, leads to complex cross-immunity dynamics at the population level, causing multi-annual cycles of outbreaks [9] which may be highly persistent [14, 15]. In Malaysia, Singapore and Thailand, all four serotypes are in active circulation, with switches in the predominantly reported serotypes over time observed in tandem with a dramatic rise in reported case counts [16–18]. Typically, people infected with dengue virus are asymptomatic or display mild symptoms such as febrile illness [19], but those infected a second or subsequent time by a different serotype are at a greater risk of more severe disease due to antibody dependent enhancement, causing potentially life-threatening conditions such as dengue hemorrhagic fever [20]. Individuals with severe dengue are provided treatment to manage clinical symptoms, but no cure exists for the virus [21]. Varying seroprevalence rates across populations also make vaccination using the tetravalent Dengvaxia (cyd-tdv) and tak-003 vaccines challenging [22], therefore, the primary public health intervention for dengue is currently vector control [21].

Intervention measures are implemented across Southeast Asia depending on the severity of the dengue outbreak observed. Cross-border surveillance systems have been set-up among the Southeast Asian countries to track plausible dengue case importation and other arboviruses [23]. Within countries, in Singapore, the management of dengue is primarily conducted through vector surveillance [24], vector control, public engagement [25] and other novel techniques such as *Wolbachia*-infected mosquitoes [26]. In Malaysia, breeding site reduction in households and water sources is conducted through domestic inspection [27] as well as larvicidal treatment [28]. The release of *Toxorhynchites* sp. mosquitoes was also conducted periodically in hotspots to reduce the vector population [29]. Similarly, Thailand applies a vector control management program to reduce breeding sites in urban and suburban locales [30]. In all three countries, control focuses on environment in which contact between mosquitoes and humans is thought to be highest, primarily residential areas [31]. In general, these interventions are also focused on localities such as Bangkok, Thailand, Kuala Lumpur, Malaysia, and Singapore, high density urban areas where transmission risk is highest, with the largest number of reported case counts [32, 33].

Social distancing (sd) measures have been broadly implemented to reduce the transmission potential of the ongoing Covid-19 pandemic [34–36]. However, despite the extant literature detailing human mobility, geographical clustering, home-work infection patterns being determinants of vector-borne diseases [9, 37–40], the effects of sd on the transmission potential of vector-borne diseases such as dengue are not known nor considered to be a standard form of intervention, due to sd’s minimal influence on vector dynamics as compared to targeted vector control measures on a localised scale. The ongoing Covid-19 pandemic has however led to population-wide implementation of sd across countries [41], with a rapidly deteriorating global situation leading to near complete lockdowns in Malaysia, Singapore and Thailand. Specifically, the reduction of movements through workplace closures and mass gatherings were implemented and heavily enforced in these countries, which have led to a large decrease in time spent in workplaces (see S1 for summary) over a period of 2 to 3 months before gradual relaxation of measures. This provides a natural experiment to estimate the effects of reducing human mobility and workplace exposure on dengue transmissions, due to the near-complete coverage of sd policies across populations and heavy compliance through enforcement of said measures [42].

We exploit the pre-post differences in human mobility and locations for exposure from sd as a quasi-experiment on reported dengue case counts across three countries in peninsular Southeast Asia, namely Malaysia, Singapore and Thailand, to estimate the treatment effects from sd on dengue transmissions. These were countries in Southeast Asia where reported dengue case counts were publicly available and recorded at the timepoints when social distancing policy was implemented. Primarily, by accounting for time-varying confounders such as climate and seasonality on the vector population [8], trends in dengue transmissions and country-specific geographical profiles, we estimate the causal effect of sd intervention by applying a regression discontinuity design (rdd) to the time point when the policy was implemented [43]. The immediate and clearly defined start date for sd policy implementation warrants the consideration of a sharp rdd rather than the fuzzy rdd of [44], which is primarily used in scenarios where the policy implementation is not prompt or when the cutoff point is not clearly defined. The treatment effect of sd policy is also applied to almost the entire population over the considered regions, with only a minimal number of essential workers allowed to work away from home and businesses to remain open. The sd policy thus encompasses a majority of each population over a large spatial scale, which minimizes the risks of substitution effects of human mobility on dengue transmissions from sd policy [45]. Compliance to the treatment is also near universal, with the primary reason being heavy enforcement by authorities, the strict penalties applied across the considered regions and individuals whom are away from home being easy to spot [46].

We first detail the data used and the rdd design considered for the three countries. Robustness checks and controlling for confounders are then conducted to ensure valid identification of sd treatment effects. The average treatment effect per month is computed for sd on dengue to look at the overall benefits attributable to sd. Finally, the implications of this study are discussed.

## Methods

### Dengue case count data

Monthly provincial dengue case data from Thailand were obtained from the National Disease Surveillance report system from January 2010 to May 2020 which is maintained by the Bureau of Epidemiology, Thailand [47]. Weekly dengue case data in Malaysia from 2010 to 2017 was obtained from Malaysia’s open data portal run by the Malaysian Administrative Modernisation and Management Planning Unit (MAMPU) [32]. The weekly dengue case data from 2018 to 2020 was extracted from the World Health Organisation Institutional Repository for Information Sharing [48]. Weekly dengue case data from 2012 to 2020 were obtained from the Weekly Infectious Diseases Bulletin published by Ministry of Health, Singapore [33]. For Singapore and Malaysia, data up until the 20^*th*^ and 14^*th*^ epidemiological week of 2020 were used respectively.

### Climate data

Climate data was obtained from ERA5, published by the European Centre for Medium-Range Weather Forecasts. ERA5 provides hourly estimates across a 30km grid [49], which we spatially averaged for all provinces of Thailand at a monthly time scale. For Singapore and Malaysia, the data is aggregated to a weekly timescale and spatially averaged. Mean temperature at 2m was calculated to represent thermal forcing and stress on vector population growth, and total rainfall as a proxy for breeding site availability. Air temperature and dewpoint temperature were utilized to calculate saturation vapor pressure and actual vapor pressure using Teten’s formula, where relative and absolute humidity could then be estimated using standard formula [50].

### Intervention data

Intervention data for social distancing were obtained through national governmental and local news websites for the start and end dates of social distancing as well as the intensity of social distancing measures implemented. The full source listing is detailed in S1.

### Population census data

Population census for Thailand by province from 2010 to 2020 was obtained from Thailand’s official statistics registrations systems which is managed by the Office of Registration Administration, Department of Local Administration. Population census data for Malaysia from 2010 to 2019 was obtained from Department of Statistics, Malaysia which contains population information by state and by country [51]. Population census data for Singapore from 2010 to 2019 was obtained from the Department of Statistics, Singapore [52]. The data and code used for this paper has been attached as supplementary material (S3).

### Identification strategy

#### Regression discontinuity design for Thailand

The goal of our analysis is to identify the policy effect of social distancing on reported dengue case counts. The sharp rdd was thus used as the primary strategy for countries of interest. Specifically for Thailand the balanced panel design across provinces was considered, due to data measured across provinces across time:

$$\begin{array}{c} y_{i,t}=\beta_{i}+{\text{I}(\text{policy})}_{t}\delta +f\left(t\right)+\sum_{j=1}^{P}\alpha_{i,j}x_{i,j,t}+\epsilon_{i,t} \end{array}$$ (1)

where *y*_*i*,*t*_ is the reported dengue case count per 10 000 individuals per province measured on the monthly frequency (bounded by 0 and 10 000) for the province *i* at time *t*, *β*_*i*_ the province specific fixed effect term to account for heterogeneous baseline risk across provinces. Confounders measured for each province *i* at the time point *t* are denoted *x*_*i*,*j*,*t*_ for *j* ∈ {1, …, *P*}. A polynomial *f*(*t*) up to order 3 accounts for the temporal dependence of observed dengue case count. The policy effect size, *δ*, is the primary estimand. I(policy)_*t*_ is an indicator variable which takes value 1 if social distancing policy is implemented and takes value 0 otherwise. Eq (1) was estimated using generalized panel least squares. Precedent studies have used the identity link function with case counts being the dependent variable and we have similarly applied it in (1) [14, 53, 54]. An order 1 autoregressive term on errors was applied to account for serial correlation between observations and a one month lag corresponds to the maximum duration for the *Ae*. *aegypti/albopictus* vector life cycle [55, 56]. Further addition of lag terms beyond one month can result in spurious correlations and invalid standard errors on the policy effect of interest [57].

#### Regression discontinuity design for Malaysia/Singapore

Due to the different frequencies and time windows measured for Malaysia and Singapore, and only nationally reported case count data available over the period of social distancing, our country specific rdd strategy for the two countries is:

$$\begin{array}{c} y_{t}=\beta +{\text{I}(\text{policy})}_{t}\delta +f\left(t\right)+\sum_{j=1}^{P}\alpha_{j}x_{j,t}+\epsilon_{t} \end{array}$$ (2)

with the *i* subscript suppressed to denote the absence of state- or district-level data, and similar notation to the Thai model. Eq (2) was estimated separately for both countries due to the different time windows when dengue case counts were collected and the different start/end dates for social distancing policy. Generalized least squares was used as the estimation strategy for each country with a 4 week lag autoregressive term on errors. This was to account for serial correlation between observations and a four week lag corresponds to the maximum duration for the *Ae*. *aegypti/albopictus* vector life cycle [55, 56]. Further addition of lag terms beyond four weeks can result in spurious correlations and invalid standard errors on the policy effect of interest [57]. The full list of confounders are described in the section below.

#### Controlling for confounders

In the rdd strategy, in addition to locality specific risks controlled using the fixed effects term, we control for province/country level confounders which may affect the transmission potential of dengue virus due to influences on vector breeding conditions and human mobility. These are summarized in Table 1.

**Table 1:** Variables controlled for in rdd identification strategy.

Country	Variable	Source
TH/SG/MS	Total Precipitation	[49]
TH/SG/MS	Dewpoint Temperature	[49]
TH/SG/MS	Absolute Humidity	[49]
TH/SG/MS	Relatively Humidity	[49]
TH	Wet/Dry Seasons	[58]
MS/SG	Monsoon/No Monsoon Season	[58]
TH/SG/MS	Time Spline: *f*(*t*) = *at* + *bt*^2^ + *ct*^3^	Derived

There is evidence that climate may affect the transmission potential of dengue virus through its effect on vector breeding potential. We account for climate confounders by including total precipitation, dewpoint temperature, absolute and relative humidity in the rdd design [59–61]. We considered one month lagged climate measurements for Thailand in each province and four week lagged climate measurements for Singapore and Malaysia to correspond to the life cycle of the *Ae*. *aegypti* and *Ae*. *albopictus* vectors to the rdd estimation. Wet/dry seasons are considered for each province of Thailand due to the savannah climate experienced in some provinces leading to distinct months where dengue transmissions are elevated [32]. Monsoon seasons are considered for Malaysia and Singapore to represent climatic forcing on vector breeding potential in these seasons [62]. Finally, the time spline *f*(*t*) was added to account for the near term transmission potential and time dependence of dengue transmissions. The time spline specification follows the large body of literature on appropriate parameterizing time-dependent measurements in rdd identification problems [63–65]. Higher order polynomial terms for the spline term were not included for model parsimony and to prevent over-fitting at the discontinuity where policy was implemented.

#### Robustness checks

We built our rdd specifications (1–2) by sequentially adding the confounders discussed in the previous section. This was conducted to ensure robust identification of the social distancing policy effect estimated in each country and to fully ensure that the policy effect estimated was not an artefact of other phenomena which affect dengue transmission periods across the periods before and after policy implementation [63]. Taking dengue case counts per 10 000 as the dependent variable, we first estimated (1–2) only using the regression discontinuity policy term. Next, seasonality was added, followed by the first to third order polynomial spline terms. Lastly, climate variables were added for additional sensitivity analysis. Sensitivity of the policy effect estimates to error term misspecification was also considered by estimating (1–2) both by ordinary least squares and the generalized least squares approach taking into account serial correlation between observations. Sensitivity of discontinuity coefficient effect size, values and standard errors to the time window of data used before the discontinuity were also checked by restricting the number of observations allowed in estimating (1–2). Multicollinearity and error normality assumptions for linear regression are checked using the variance inflation factor and quantile-quantile plots. The full output for these robustness checks are provided in S2.

## Results

### Trends in dengue cases across countries

**Fig 1:**
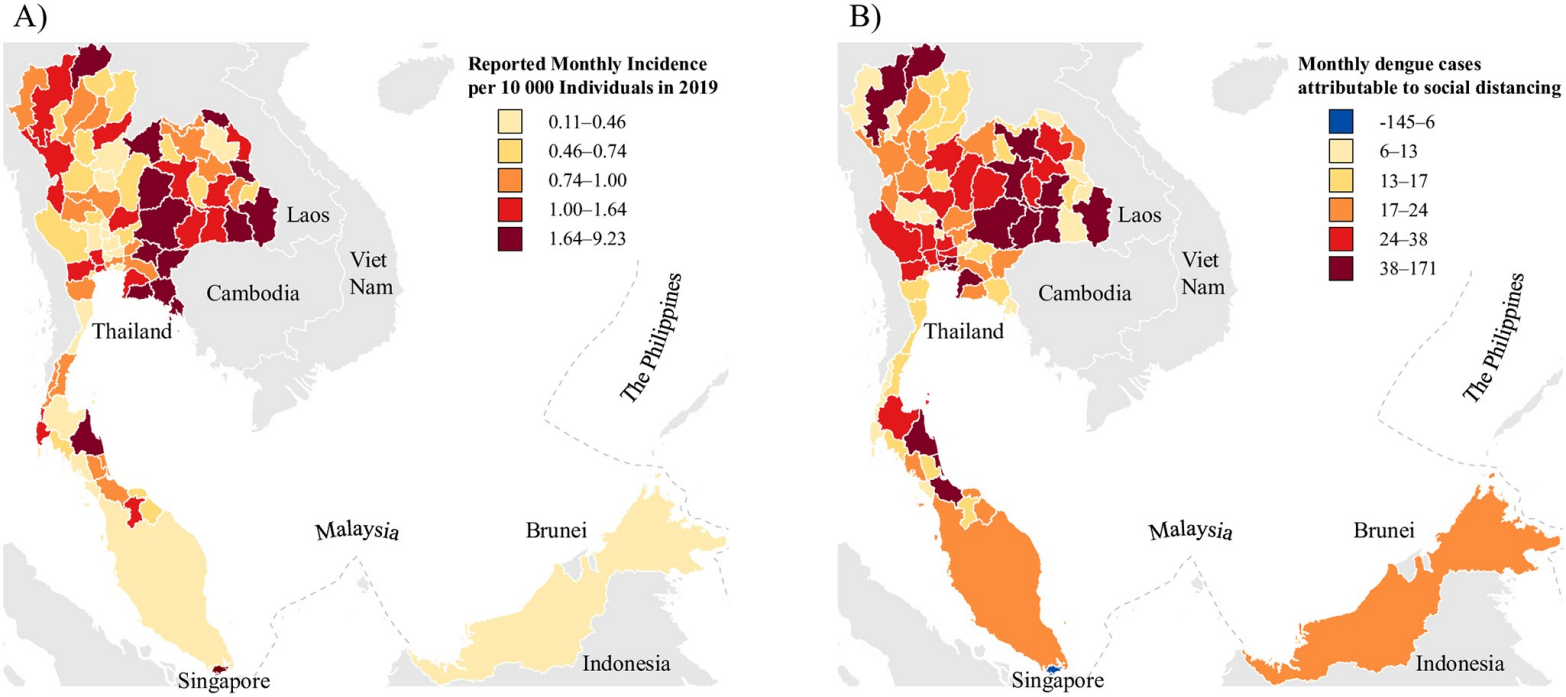
A) Reported dengue cases per 10 000 in 2019 for all provinces of Thailand and Singapore and Malaysia B) Expected number of dengue case counts averted/gained per month for all provinces of Thailand, and nationally for Malaysia and Singapore.

The average number of reported dengue cases per 10 000 in 2019 across all provinces of Thailand is 1.15 cases per 10 000 persons. The province with the lowest rate of reported dengue cases is Sing Buri at 0.11 cases per 10 000 persons while the highest reported dengue cases per 10 000 across Thailand is incurred by Si Sa Ket at 9.23 cases per 10 000 persons. The number of dengue cases was found to be 2.15 cases per 10 000 persons in Singapore, which was the highest number of dengue cases across the three countries. In Malaysia, the number of dengue cases was found to be 0.31 cases per 10 000 persons (Fig 1).

### Heterogeneous effects of social distancing on dengue cases across countries

The policy effect of social distancing in Thailand is positive. With implementation of social distancing, the number of dengue cases in Thailand is expected to increase by 0.807 cases per 10 000 people when not controlling for seasonality or time trend (Table 2, Model 1). After adding additional variables controlling for seasonality climate and the time trend, the number of dengue cases in Thailand is expected to increase by 0.431 cases per 10 000 people per province after implementation of social distancing keeping other confounders fixed. Even with additional variables that were added in subsequent models (Table 2), the coefficients for the policy effect on dengue cases per 10 000 people continues to remain positive and statistically significant (*p* < 0.001), illustrating that the policy effect of social distancing increases the number of dengue cases within Thailand. With the implementation of social distancing, the absolute increase in dengue cases per month on average in each province is expected to be 26.1 (95% CI: 15.2 to 36.9). The highest increase in number of dengue cases due to sd is attributable to Bangkok at an expected 171.0 (95% CI: 99.6 to 242.3). The lowest increase in dengue cases is incurred due to sd policy by Samut Sakhon at an expected 5.8 (95% CI: 3.4 to 8.3).

**Table 2:** Policy effect of sd on dengue incidence in Thailand. Estimation was conducted using the panel generalized least squares procedure taking into account serial correlation and between province correlations on our dependent variable of up to one month. Numbers in parenthesis refer to standard error of the coefficients.

Dependent Variable: Dengue cases Per 10 000					
	(1)	(2)	(3)	(4)	(5)
Policy	0.80697***	0.35564***	0.38408***	0.30174***	0.43176*
	(0.01617)	(0.02106)	(0.04007)	(0.06423)	(0.19193)
I(season)		−0.64239***	−0.64181***	−0.64386***	−0.45102***
		(0.00994)	(0.01013)	(0.01050)	(0.00247)
*T*			−0.00032***	−0.00342***	−0.00233***
			(0.00004)	(0.00010)	(0.00021)
*T*^2^				0.00003***	0.00001***
				(0.00000)	(0.00000)
Absolute Humidity Lag 1					0.15019***
					(0.00023)
Total Precipitation Lag 1					−0.38286***
					(0.00165)
Relative Humidity Lag 1					−0.02235***
					(0.00006)
Average Temperature Lag 1					−0.00830***
					(0.00023)
Num. obs.	8997	8997	8997	8997	8997

***p < 0.001,

**p < 0.01,

*p < 0.05,

^⋅^*p* < 0.1

Social distancing policy did not have a statistically significant effect on the number of dengue cases in Singapore. The policy effect was expected to increase the number of dengue cases in Singapore by 2.15 cases per 10 000 persons. The coefficients are positive but addition of seasonality or a time trend did not result in policy effects which 95% confidence intervals are away from 0 across all 5 models. The largest policy effect was found to be 0.06201 in model 2 (Table 3) but was not found to be statistically significant. The smallest policy effect was found to be 0.03558 in model 5 (Table 3) but was also not found to be statistically significant. The coefficients were not statistically significant after addition of confounding factors such as climate variables. The number of dengue cases averted due to sd is expected to be at 21.03 (95% CI: -65.64 to 107.70) per 10 000 individuals but with 95% confidence interval containing zero.

**Table 3:** Policy effect of sd on dengue cases in Singapore. Estimation was conducted using the feasible generalized least squares procedure taking into account serial correlation of our dependent variable of up to four weeks. Numbers in parenthesis refer to standard error of the coefficients.

Dependent Variable: Dengue Cases Per 10000					
	(1)	(2)	(3)	(4)	(5)
(Intercept)	0.41850***	0.41154***	0.38240*	0.44897	4.25056
	(0.09065)	(0.09048)	(0.18920)	(0.28518)	(3.97952)
Policy	0.06201	0.06166	0.05872	0.05414	0.03558
	(0.07748)	(0.07752)	(0.07779)	(0.07813)	(0.07717)
I(season)		0.01365	0.01339	0.01319	0.01200
		(0.01901)	(0.01902)	(0.01902)	(0.01886)
*T*			0.00014	−0.00095	0.01372***
			(0.00073)	(0.00300)	(0.00344)
*T*^2^				0.00000	−0.00008***
				(0.00001)	(0.00002)
*T*^3^					0.00000***
					(0.00000)
Average Temperature Lag 1					−0.28571
					(0.24354)
Total Precipitation Lag 1					0.01841
					(0.01937)
Absolute Humidity Lag 1					0.29930
					(0.23626)
Relative Humidity Lag 1					−0.06713
					(0.05449)
Num. obs.	437	437	437	437	437

***p < 0.001,

**p < 0.01,

*p < 0.05,

^⋅^*p* < 0.1

The sd policy was also not found to significantly affect number of dengue cases per 10 000 individuals in Malaysia. The coefficients are negative but not statistically significant across all 5 models considered. The least negative coefficient was seen in model 5 at -0.00398 (Table 4) while the most negative coefficient was seen in model 3 at -0.00772 (Table 4) but both were found to be not statistically significant. Similar to Singapore, the addition of seasonality or time trend did not result in a significant policy effect on dengue cases in Malaysia. The expected number of dengue cases averted due to sd is 145.32 (95% CI: -617.07 to 326.43), however this 95% confidence interval contains zero.

**Table 4:** Policy effect of sd on dengue cases in Malaysia. Estimation was conducted using the feasible generalized least squares procedure taking into account serial correlation of our dependent variable of up to four weeks. Numbers in parenthesis refer to standard error of the coefficients.

Dependent Variable: Dengue Cases Per 10000					
	(1)	(2)	(3)	(4)	(5)
(Intercept)	0.05898***	0.05933***	0.04352**	0.02177	1.54397*
	(0.01020)	(0.01055)	(0.01331)	(0.01419)	(0.77346)
Policy	−0.00610	−0.00615	−0.00772	−0.00603	−0.00398
	(0.00724)	(0.00727)	(0.00732)	(0.00731)	(0.00749)
I(season)		−0.00051	−0.00060	0.00015	0.00075
		(0.00367)	(0.00366)	(0.00361)	(0.00360)
*T*			0.00030	0.00143**	−0.00001
			(0.00019)	(0.00054)	(0.00142)
*T*^2^				−0.00001*	0.00002
				(0.00000)	(0.00003)
*T*^3^					−0.00000
					(0.00000)
Average Temperature Lag 1					−0.09193^⋅^
					(0.04776)
Total Precipitation Lag 1					0.01015
					(0.00784)
Absolute Humidity Lag 1					0.09014^⋅^
					(0.04590)
Relative Humidity Lag 1					−0.02103*
					(0.01045)
Num. obs.	118	118	118	118	118

***p < 0.001,

**p < 0.01,

*p < 0.05,

^⋅^*p* < 0.1

### Robustness checks

The policy effect of sd on number of dengue cases per 10 000 in Thailand remains positive and statistically significant at the 0.001% level even under sensitivity analysis of our rdd design to model misspecification or omitting biologically important covariates. We conducted exercises by omitting either serial correlation between provinces or within province correlations in the panel generalized least squares procedure, shortening the time period of data for estimation, or the addition/omission of one month lag climatic variables but no change in direction nor statistical significance was observed (S2). Similarly, the policy effect of sd on number of dengue cases per 10 000 in Singapore and Malaysia has 95% confidence intervals containing 0 under sensitivity analysis of our rdd design to model misspecification or omitting biologically important covariates. However, one instance where the 95% confidence intervals excluded 0 was when serial correlation was omitted and no time dependence between successive dengue case count observations were taken into account yielded a negative sd policy effect on dengue cases per 10 000 in Malaysia at -0.03048 and negative or positive sd policy effect on dengue cases per 10 000 in Singapore. These estimates however were sensitive to addition or omission of covariates and did not take into account serial correlation of the dependent variable.

## Discussion

Results indicate that implementation of sd has led to an increase in dengue cases in Thailand, as compared to Singapore and Malaysia where this effect was not found. One key pathway that may explain this result is the propensity for dengue infections to surface at home rather than work addresses, and the disparity in sd policy effects being driven by across country differences in residential structure. Implementation of sd policies result in an increase in time spent within home addresses and thus increased risk of dengue infections if infections mostly occur at the home. Although it is possible for dengue infections to occur in workplaces, it was found in one study that 60% of dengue cases live less than 200m apart came from the same transmission chain, revealing that residential areas are a focal point of transmission [31]. Additionally, an increased frequency of movement within urban and predominantly residential neighbourhoods was found to increase the risk of dengue infection [66]. Patterns in urban area structure and population density could also influence the rate of dengue incidence, as agglomerations of high-rise buildings have a lower dengue incidence as compared to low-rise buildings [67]. Urban and rural areas with predominantly low-rise residential buildings with denser drainage networks could thus increase the breeding habitats of dengue vectors. [67]. While large differences in urbanisation demographics may potentially affect dengue preventive practices especially in Malaysia and Thailand with significant rural populations [68], multiple studies have shown that there is no large difference in dengue incidences between urban and rural areas in either Malaysia [69, 70] or Thailand [71, 72]. Also, spatio-temporal studies of countries with similar climate comparing dengue incidence between urban and rural also found no significant difference in dengue incidence between study areas [73, 74]. Thus, in terms of geographical confounding, the disparities in sd policy effects on dengue incidence between these countries is likely to be driven by across country differences in residential structure rather than differences in urban-rural structure [75].

Serotype switching is well studied as a possible cause of dengue outbreaks in dengue endemic regions such as Singapore, Malaysia and Thailand [16, 76]. This is not explicitly accounted for in the rdd design due to data availability issues. In Singapore, a sustained switch in the predominant serotype from denv-1/denv-2 to denv-3 has led to a drastic rise in dengue case counts in 2020 [77]. The inception of serotype switching could influence reported case counts by providing temporary or partial cross immunity that is passed onto others within the community [17, 78], but these population level dynamics were not explicitly accounted for in our rdd design. However, switches to/ from denv-1 and denv-2 have been linked to large outbreaks in Singapore have also suggested contemporaneously low population immunity to these strains [16]. Currently, immunity to denv-3 is even lower as it has not been the predominant serotype in Singapore’s dengue history until recent months [77]. These effects may have confounded the effect of social distancing on dengue incidence in Singapore, but given that the transmission leadership of denv-3 has not been observed in the past, it is difficult to determine the direction of effect on reported case counts. Conversely, the phenomenon of serotype switching has not been reported for Thailand or Malaysia around the time where sd is implemented, which discounts the possible effect of cross-immunity dynamics on reported case counts in these populations. Other long term effects from cross immunity dynamics such as changing sero-prevalence and source-sink importations are also expressed in multi-annual cycles [9, 17], which is unlikely to bias estimates of the discontinuity at the point of social distancing in either direction for Thailand and Malaysia.

In Thailand, we estimate an additional number of dengue cases per month to be 2008.34 (95% CI: 1170.40 to 2846.28) due to social distancing policy, primarily through increased exposure in residences across the country as discussed. However, as the genetic diversity of dengue around one’s home is likely to remain fixed in absence of locality specific importation, recovery from infection from a serotype confers life-long immunity to that serotype [17] which may strengthened the herd immunity effect in each neighborhood, leading to a gradual decrease in the rise of dengue cases over a longer period of time should sd policies persist. Increases in mixing patterns away from home by exiting from sd policy however, may lead to an increased risk of locality specific importation into residences. With countries gradually easing social distancing policies and dengue exposure patterns shifting to the pre-sd policy regime (See S1 for policy summary), the future impact of exiting from sd policy on dengue transmissions is unknown and necessitates further study. However, should sd policy be re-instituted, vector control in residential areas is necessary to reduce the transmission potential of dengue [79].

Social distancing policy is used as a public health intervention for respiratory diseases as it reduces human contact by increasing time spent in residences and a reduction in time spent elsewhere. This decreases the transmission potential for these diseases by changing the mixing patterns within the host population [35, 80]. sd policies motivated by the sars-cov-2 pandemic also reduced the transmission potential of sars-cov-2 as well as other respiratory illnesses [81, 82]. A change in human movement patterns can likewise influence the transmission pattern of vector-borne diseases, as evidenced from this study—primarily through changing the host risk of exposure to vectors, but in the opposing direction. More time spent at a site was shown to increase transmission risk of vector-borne diseases irregardless of vector density [83]. The transmission risk of vector-borne diseases could be higher at home and in one’s neighbourhood, such as with dengue [31], malaria [84] and Chikungunya [85]. Social distancing and increasing time spent at residences could lead to higher incidence of vector-borne diseases, which corroborates the policy effect seen for Thailand. It is of interest to generalize the natural experiment design of social distancing on other vector-borne diseases as the policy effect on the reported incidence of other diseases may be different. The *Aedes* mosquito vector, which transmits dengue, Chikungunya, Zika virus and yellow fever prefer breeding in artificial containers around the house or holes in trees, while the *Anopheles* mosquito vector, which transmits malaria, prefer permanent water features such as lakes [86]. The differences in habitats for these vectors may result in differences in exposure risk pre-post policy for the host population and further work is needed to ascertain the sd policy effect on these other vector-borne diseases.

This study identifies the pre-post sd policy effects on dengue incidence while controlling for biologically important covariates which vary across time, such as seasonality, climate and time trend. Robustness checks also reveal the persistence of our results under omission/addition of these variables, with or without model misspecification. The heavy enforcement and compliance to sd policy measures on a set start date across the three countries studied also mean that treatment effects are likely to apply to a large proportion of each population and the sharp rdd is appropriate. There are however a few limitations to our study. The implementation of social distancing policies could result in the under reporting of dengue cases as individuals may be less willing to leave their homes and seek professional medical treatment. Under reporting may also result from the additional burden sars-cov-2 places on health systems, which decreases the number of non-covid-19 patients seeking treatment This could bias downwards the estimated sd policy effect on the incidence of dengue. When more data become available, compartmental models could be used to adjust for the reporting rate. Additionally, cross-immunity dynamics of dengue in populations are difficult to account for, however, we reason that these are likely long term trends which are unlikely to affect short term discontinuities generated by the sd policy. The lack of available spatial data for Malaysia and Singapore means that the rdd design is conducted at a national scale, which may not be able to account for differences in pre-post policy spatio-temporal effects on dengue transmission, though for both countries, the policy implemented was a national one so the impact of this should be limited.

## Supporting information

S1 Appendix (PDF)

S2 Appendix (DOCX)
