# Supplementary material for: Impact of sars-cov-2 interventions on dengue transmission
Source: PLoS Negl Trop Dis. 2020 Oct 29;14(10):e0008719. doi: 10.1371/journal.pntd.0008719 (PMC7595279; doi:10.1371/journal.pntd.0008719)
Supplement: S1 Appendix — (PDF) [file pntd.0008719.s001.pdf]

Technical Appendix 1

July 7, 2020

Contents

1 Robustness Check: Pooled OLS (Thailand) 2

2 Robustness Check: Panel OLS with Province Fixed Effects (Thailand) 2

3 Robustness Check: Panel FGLS with Province Fixed Effects and Robust SE (Thailand) 3
- 3.1 Restrict data to 48 months before SD . . . 3
- 3.2 Restrict data to 60 months before SD . . . 3

4 Robustness Check: OLS not taking into account serial correlation (Malaysia) 4

5 Robustness Check: FGLS taking into account serial correlation (Malaysia) 4
- 5.1 Restrict data to 52 Weeks . . . 4
- 5.2 Restrict data to 77 Weeks . . . 5
- 5.3 Full Model with climate variables . . . 6

6 Robustness Check: OLS not taking into account serial correlation (Singapore) 7

7 Robustness Check: FGLS taking into account serial correlation (Singapore) 7
- 7.1 Restrict data to 52 Weeks . . . 7
- 7.2 Restrict data to 77 Weeks . . . 8
- 7.3 Full Model with Climate Variables . . . 9

8 Robustness Check: Variance Inflation Factor 10

9 Robustness Check: Quantile-quantile plots 11

1 Robustness Check: Pooled OLS (Thailand)

	Model 1	Model 2	Model 3	Model 4	Model 5
(Intercept)	0.76556***	1.20849***	0.78118***	0.80617***	0.52104***
	(0.01636)	(0.02828)	(0.03293)	(0.04993)	(0.06745)
Policy	0.79951***	0.35657**	0.81526***	0.78967***	0.49190***
	(0.12505)	(0.12481)	(0.12834)	(0.13397)	(0.14186)
I(Season)		−0.65303***			
		(0.03434)			
T			−0.00027	−0.00155	0.02733***
			(0.00049)	(0.00199)	(0.00501)
T^2				0.00001	−0.00061***
				(0.00002)	(0.00010)
T^3					0.00000***
					(0.00000)
AIC	33288.71376	32936.08948	33290.41493	33291.97121	33254.70470
BIC	33310.02770	32964.50807	33318.83351	33327.49445	33297.33258
Log Likelihood	-16641.35688	-16464.04474	-16641.20746	-16640.98561	-16621.35235
Deviance	21291.89604	20468.98261	21291.18885	21290.13884	21197.42261
Num. obs.	8997	8997	8997	8997	8997

$^{***}p < 0.001$, $^{**}p < 0.01$, $^{*}p < 0.05$, $^{\cdot}p < 0.1$

Table 1

2 Robustness Check: Panel OLS with Province Fixed Effects (Thailand)

	Model 1	Model 2	Model 3	Model 4
policy	0.97743***	0.57836***	0.56477***	0.48046***
	(0.00388)	(0.05706)	(0.10378)	(0.10401)
I(Season)		−0.61852***	−0.61768***	−0.61975***
		(0.00421)	(0.00490)	(0.00609)
T			−0.00031***	−0.00682***
			(0.00003)	(0.00016)
T^2				0.00005***
				(0.00000)
R^2	0.16853	0.20145	0.20148	0.20187
Num. obs.	7304	7304	7304	7304

$^{***}p < 0.001$, $^{**}p < 0.01$, $^{*}p < 0.05$, $^{\cdot}p < 0.1$

Table 2

3 Robustness Check: Panel FGLS with Province Fixed Effects and Robust SE (Thailand)

3.1 Restrict data to 48 months before SD

	Model 1	Model 2	Model 3	Model 4
	0.91357***	0.63958***	0.47583***	−0.17751***
	(0.03949)	(0.04007)	(0.03475)	(0.03453)
I(Season)		−0.39936***	−0.40058***	−0.43847***
		(0.02116)	(0.02182)	(0.02472)
T			0.00671***	−0.28331***
			(0.00046)	(0.01438)
T^2				0.00157***
				(0.00008)
R^2	0.29298	0.32510	0.33259	0.38817
Num. obs.	3608	3608	3608	3608

$^{***}p < 0.001$, $^{**}p < 0.01$, $^{*}p < 0.05$, $^{\cdot}p < 0.1$

Table 3

3.2 Restrict data to 60 months before SD

	Model 1	Model 2	Model 3	Model 4
Policy	0.97743***	0.57836***	0.56477***	0.48046***
	(0.00388)	(0.05706)	(0.10378)	(0.10401)
I(Season)		−0.61852***	−0.61768***	−0.61975***
		(0.00421)	(0.00490)	(0.00609)
T			−0.00031***	−0.00682***
			(0.00003)	(0.00016)
T^2				0.00005***
				(0.00000)
R^2	0.16853	0.20145	0.20148	0.20187
Num. obs.	7304	7304	7304	7304

$^{***}p < 0.001$, $^{**}p < 0.01$, $^{*}p < 0.05$, $^{\cdot}p < 0.1$

Table 4

4 Robustness Check: OLS not taking into account serial correlation (Malaysia)

	Model 1	Model 2	Model 3	Model 4	Model 5
(Intercept)	0.06461^{***}	0.06636^{***}	0.03960^{***}	0.02746^{***}	0.02760^{***}
	(0.00201)	(0.00328)	(0.00326)	(0.00481)	(0.00604)
Policy	−0.01438	−0.01332	-0.04111^{***}	-0.03065^{***}	-0.03048^{**}
	(0.01091)	(0.01105)	(0.00798)	(0.00827)	(0.00935)
I(Season)		−0.00281	−0.00407	−0.00137	−0.00137
		(0.00416)	(0.00286)	(0.00286)	(0.00287)
T			0.00048^{***}	0.00102^{***}	0.00101^{*}
			(0.00004)	(0.00017)	(0.00041)
T^2				-0.00000^{**}	−0.00000
				(0.00000)	(0.00001)
T^3					−0.00000
					(0.00000)
AIC	-567.79906	-566.26617	-653.86589	-662.86178	-660.86341
BIC	-559.48701	-555.18343	-640.01246	-646.23767	-641.46862
Log Likelihood	286.89953	287.13309	331.93294	337.43089	337.43171
Deviance	0.05340	0.05319	0.02489	0.02268	0.02268
Num. obs.	118	118	118	118	118

$^{***}p < 0.001$, $^{**}p < 0.01$, $^{*}p < 0.05$, $^{\cdot}p < 0.1$

Table 5

5 Robustness Check: FGLS taking into account serial correlation (Malaysia)

5.1 Restrict data to 52 Weeks

	Model 1	Model 2	Model 3	Model 4	Model 5
(Intercept)	0.07098^{***}	0.07530^{***}	0.08663	−0.46130	2.03944
	(0.00923)	(0.00959)	(1.99055)	(1.34448)	(3.03220)
Policy	−0.00703	−0.00710	−0.00511	−0.00303	−0.00080
	(0.00834)	(0.00830)	(0.00860)	(0.00867)	(0.00867)
I(Season)		−0.00660	−0.00602	−0.00524	−0.00625
		(0.00588)	(0.00601)	(0.00600)	(0.00597)
T			−0.00040	0.01255	−0.07388
			(0.00165)	(0.01053)	(0.07844)
T^2				−0.00007	0.00090
				(0.00006)	(0.00087)
T^3					−0.00000
					(0.00000)
AIC	-313.35015	-304.12812	-289.35581	-271.18922	-246.83769
BIC	-300.10741	-289.15851	-272.70448	-252.90281	-226.96440
Log Likelihood	163.67508	160.06406	153.67790	145.59461	134.41884
Num. obs.	51	51	51	51	51

$^{***}p < 0.001$, $^{**}p < 0.01$, $^{*}p < 0.05$, $^{\cdot}p < 0.1$

Table 6

5.2 Restrict data to 77 Weeks

	Model 1	Model 2	Model 3	Model 4	Model 5
(Intercept)	0.07219***	0.07715***	0.07360**	−0.06423	0.16600
	(0.00615)	(0.00729)	(0.02581)	(0.11111)	(1.76062)
Policy	−0.00711	−0.00703	−0.00694	−0.00410	−0.00199
	(0.00801)	(0.00796)	(0.00818)	(0.00835)	(0.00850)
I(Season)		−0.00679	−0.00676	−0.00577	−0.00612
		(0.00550)	(0.00563)	(0.00574)	(0.00573)
T			0.00003	0.00394	−0.00737
			(0.00031)	(0.00300)	(0.02036)
T^2				−0.00003	0.00014
				(0.00002)	(0.00027)
T^3					−0.00000
					(0.00000)
AIC	-491.50244	-482.42400	-465.91699	-445.45899	-418.06768
BIC	-475.28002	-463.99148	-445.30285	-422.69233	-393.17820
Log Likelihood	252.75122	249.21200	241.95849	232.72950	220.03384
Num. obs.	77	77	77	77	77

$^{***}p < 0.001$, $^{**}p < 0.01$, $^{*}p < 0.05$, $^{\cdot}p < 0.1$

Table 7

5.3 Full Model with climate variables

	Model 1	Model 2	Model 3	Model 4
(Intercept)	1.54397*	3.31166*	3.63443$^{\cdot}$	6.24377*
	(0.77346)	(1.32391)	(2.04191)	(2.67542)
Policy	−0.00398	−0.00268	−0.00762	−0.00900
	(0.00749)	(0.00728)	(0.00795)	(0.00821)
T	−0.00001	0.00006	0.00025	0.00018
	(0.00142)	(0.00142)	(0.00149)	(0.00129)
T^2	0.00002	0.00002	0.00002	0.00002
	(0.00003)	(0.00003)	(0.00003)	(0.00003)
T^3	−0.00000	−0.00000	−0.00000	−0.00000
	(0.00000)	(0.00000)	(0.00000)	(0.00000)
Average Temperature Lag 1	−0.09193$^{\cdot}$	−0.12828*	−0.13704*	−0.15991**
	(0.04776)	(0.05043)	(0.05367)	(0.05479)
Total Precipitation Lag 1	0.01015	0.00599	0.00411	0.00563
	(0.00784)	(0.00819)	(0.00893)	(0.00921)
Absolute Humidity Lag 1	0.09014$^{\cdot}$	0.12413*	0.13357*	0.15389**
	(0.04590)	(0.04854)	(0.05153)	(0.05253)
Relative Humidity Lag 1	−0.02103*	−0.02858*	−0.03088**	−0.03558**
	(0.01045)	(0.01100)	(0.01171)	(0.01195)
I(Season)	0.00075	0.00121	0.00085	−0.00232
	(0.00360)	(0.00348)	(0.00354)	(0.00366)
Average Temperature Lag 2		−0.07487	−0.07671	−0.09797
		(0.05249)	(0.05889)	(0.06112)
Total Precipitation Lag 2		0.00257	−0.00468	−0.00003
		(0.00785)	(0.00888)	(0.00962)
Absolute Humidity Lag 2		0.07502	0.07486	0.09396
		(0.05060)	(0.05693)	(0.05883)
Relative Humidity Lag 2		−0.01718	−0.01719	−0.02145
		(0.01147)	(0.01283)	(0.01333)
Average Temperature Lag 3			−0.00637	−0.04407
			(0.05588)	(0.06019)
Total Precipitation Lag 3			−0.00986	0.00188
			(0.00918)	(0.00999)
Absolute Humidity Lag 3			0.00728	0.04513
			(0.05372)	(0.05800)
Relative Humidity Lag 3			−0.00203	−0.01043
			(0.01220)	(0.01313)
Average Temperature Lag 4				−0.08044
				(0.05782)
Total Precipitation Lag 4				0.00999
				(0.00901)
Absolute Humidity Lag 4				0.07427
				(0.05561)
Relative Humidity Lag 4				−0.01644
				(0.01262)
AIC	-676.83999	-638.14482	-599.61392	-565.06660
BIC	-636.60802	-587.90139	-539.69500	-495.82920
Log Likelihood	353.41999	338.07241	322.80696	309.53330
Num. obs.	118	118	118	118

$^{***}p < 0.001$, $^{**}p < 0.01$, $^{*}p < 0.05$, $^{\cdot}p < 0.1$

Table 8

6 Robustness Check: OLS not taking into account serial correlation (Singapore)

	Model 1	Model 2	Model 3	Model 4	Model 5
(Intercept)	0.40448***	0.37034***	0.45133***	0.47557***	−0.10159*
	(0.01527)	(0.02147)	(0.03432)	(0.04796)	(0.04667)
Policy	0.33161*	0.29731*	0.37922**	0.35345*	−0.26489*
	(0.13034)	(0.13062)	(0.13226)	(0.13704)	(0.10632)
I(Season)		0.06844*	0.06396*	0.06457*	0.08291***
		(0.03040)	(0.03016)	(0.03019)	(0.02232)
T			−0.00036**	−0.00071	0.01497***
			(0.00012)	(0.00049)	(0.00090)
T^2				0.00000	−0.00009***
				(0.00000)	(0.00000)
T^3					0.00000***
					(0.00000)
AIC	240.20357	237.13106	230.09301	231.56306	-32.27293
BIC	252.44337	253.45079	250.49268	256.04266	-3.71340
Log Likelihood	-117.10179	-114.56553	-110.04651	-109.78153	23.13647
Deviance	43.72811	43.22346	42.33870	42.28738	23.01551
Num. obs.	437	437	437	437	437

$^{***}p < 0.001$, $^{**}p < 0.01$, $^{*}p < 0.05$, $^{\cdot}p < 0.1$

Table 9

7 Robustness Check: FGLS taking into account serial correlation (Singapore)

7.1 Restrict data to 52 Weeks

	Model 1	Model 2	Model 3	Model 4	Model 5
(Intercept)	0.68023***	0.62937***	1.11785	59.03153	−2202.02057
	(0.07577)	(0.06240)	(1.67695)	(46.96951)	(1552.71092)
Policy	0.05798	0.05438	0.05861	0.04688	0.03245
	(0.07322)	(0.07170)	(0.07434)	(0.07456)	(0.07483)
I(Season)		0.09020·	0.08396	0.06769	0.06047
		(0.04995)	(0.05096)	(0.05256)	(0.05267)
T			−0.00118	−0.28285	16.24230
			(0.00408)	(0.22881)	(11.35525)
T^2				0.00034	−0.03988
				(0.00028)	(0.02766)
T^3					0.00003
					(0.00002)
AIC	-100.06902	-96.61275	-85.46918	-70.29001	-50.58633
BIC	-86.82628	-81.64314	-68.81785	-52.00359	-30.71304
Log Likelihood	57.03451	56.30637	51.73459	45.14500	36.29316
Num. obs.	51	51	51	51	51

$^{***}p < 0.001$, $^{**}p < 0.01$, $^{*}p < 0.05$, $^{\cdot}p < 0.1$

Table 10

7.2 Restrict data to 77 Weeks

	Model 1	Model 2	Model 3	Model 4	Model 5
(Intercept)	0.54739^{***}	0.53029^{***}	-2.28993	-11.07231	-503.49507
	(0.12658)	(0.12222)	(1.39937)	(27.48361)	(566.83293)
Policy	0.05567	0.05432	0.04180	0.04424	0.03691
	(0.07049)	(0.07078)	(0.07025)	(0.07123)	(0.07133)
I(Season)		0.03429	0.03388	0.03209	0.02621
		(0.04108)	(0.04043)	(0.04073)	(0.04101)
T			0.00709^{*}	0.05120	3.79213
			(0.00351)	(0.13855)	(4.29051)
T^2				-0.00006	-0.00951
				(0.00017)	(0.01080)
T^3					0.00001
					(0.00001)
AIC	-165.68196	-159.79386	-151.92296	-134.48388	-111.75841
BIC	-149.45954	-141.36134	-131.30883	-111.71722	-86.86893
Log Likelihood	89.84098	87.89693	84.96148	77.24194	66.87921
Num. obs.	77	77	77	77	77

$^{***}p < 0.001$, $^{**}p < 0.01$, $^{*}p < 0.05$, $^{\cdot}p < 0.1$

Table 11

7.3 Full Model with Climate Variables

	Model 1	Model 2	Model 3	Model 4
(Intercept)	4.25056	−4.91781	−21.65994*	−42.07708**
	(3.97952)	(6.80232)	(9.90692)	(14.05078)
Policy	0.03558	0.03111	0.03330	0.02813
	(0.07717)	(0.07679)	(0.07623)	(0.07617)
T	0.01372***	0.01375***	0.01375***	0.01380***
	(0.00344)	(0.00334)	(0.00323)	(0.00311)
T^2	−0.00008***	−0.00008***	−0.00008***	−0.00008***
	(0.00002)	(0.00002)	(0.00002)	(0.00002)
T^3	0.00000***	0.00000***	0.00000***	0.00000***
	(0.00000)	(0.00000)	(0.00000)	(0.00000)
Average Temperature Lag 1	−0.28571	−0.15925	−0.00073	0.18957
	(0.24354)	(0.25660)	(0.26476)	(0.27746)
Total Precipitation Lag 1	0.01841	0.01670	0.01422	0.00677
	(0.01937)	(0.02029)	(0.02106)	(0.02205)
Absolute Humidity Lag 1	0.29930	0.17731	0.02920	−0.15159
	(0.23626)	(0.24925)	(0.25707)	(0.26938)
Relative Humidity Lag 1	−0.06713	−0.03933	−0.00536	0.03629
	(0.05449)	(0.05748)	(0.05937)	(0.06218)
I(Season)	0.01200	0.00958	0.01407	0.01013
	(0.01886)	(0.01887)	(0.01871)	(0.01877)
Average Temperature Lag 2		0.43149·	0.67094*	0.89216**
		(0.25833)	(0.27438)	(0.29743)
Total Precipitation Lag 2		0.01092	0.00127	−0.00716
		(0.02018)	(0.02124)	(0.02312)
Absolute Humidity Lag 2		−0.40053	−0.63505*	−0.84284**
		(0.25074)	(0.26667)	(0.28893)
Relative Humidity Lag 2		0.09122	0.14410*	0.19275**
		(0.05784)	(0.06153)	(0.06678)
Average Temperature Lag 3			0.64100*	0.88938**
			(0.26748)	(0.29798)
Total Precipitation Lag 3			−0.01594	−0.02741
			(0.02088)	(0.02308)
Absolute Humidity Lag 3			−0.60873*	−0.84871**
			(0.25943)	(0.28944)
Relative Humidity Lag 3			0.13752*	0.19318**
			(0.05994)	(0.06691)
Average Temperature Lag 4				0.56470*
				(0.28046)
Total Precipitation Lag 4				−0.01293
				(0.02184)
Absolute Humidity Lag 4				−0.52950·
				(0.27198)
Relative Humidity Lag 4				0.12288·
				(0.06280)
AIC	-868.99158	-843.93522	-824.16994	-799.09747
BIC	-808.13982	-767.03515	-731.29891	-690.33395
Log Likelihood	449.49579	440.96761	435.08497	426.54873
Num. obs.	437	437	437	437

$^{***}p < 0.001$, $^{**}p < 0.01$, $^{*}p < 0.05$, $^{\cdot}p < 0.1$

Table 12

8 Robustness Check: Variance Inflation Factor

Covariate	Singapore	Thailand	Malaysia
Policy	1.06	1.09	1.13
Time Trend	1.04	1.05	1.11
Seasonality	1.02	1.04	1.02
Average Temperature	3843.32	1.55	2708.78
Absolute Humidity	2612.25	2.46	2538.46
Total Precipitation	1.47	1.49	1.98
Relative Humidity	1896.88	1.93	1861.55

Table 13

9 Robustness Check: Quantile-quantile plots

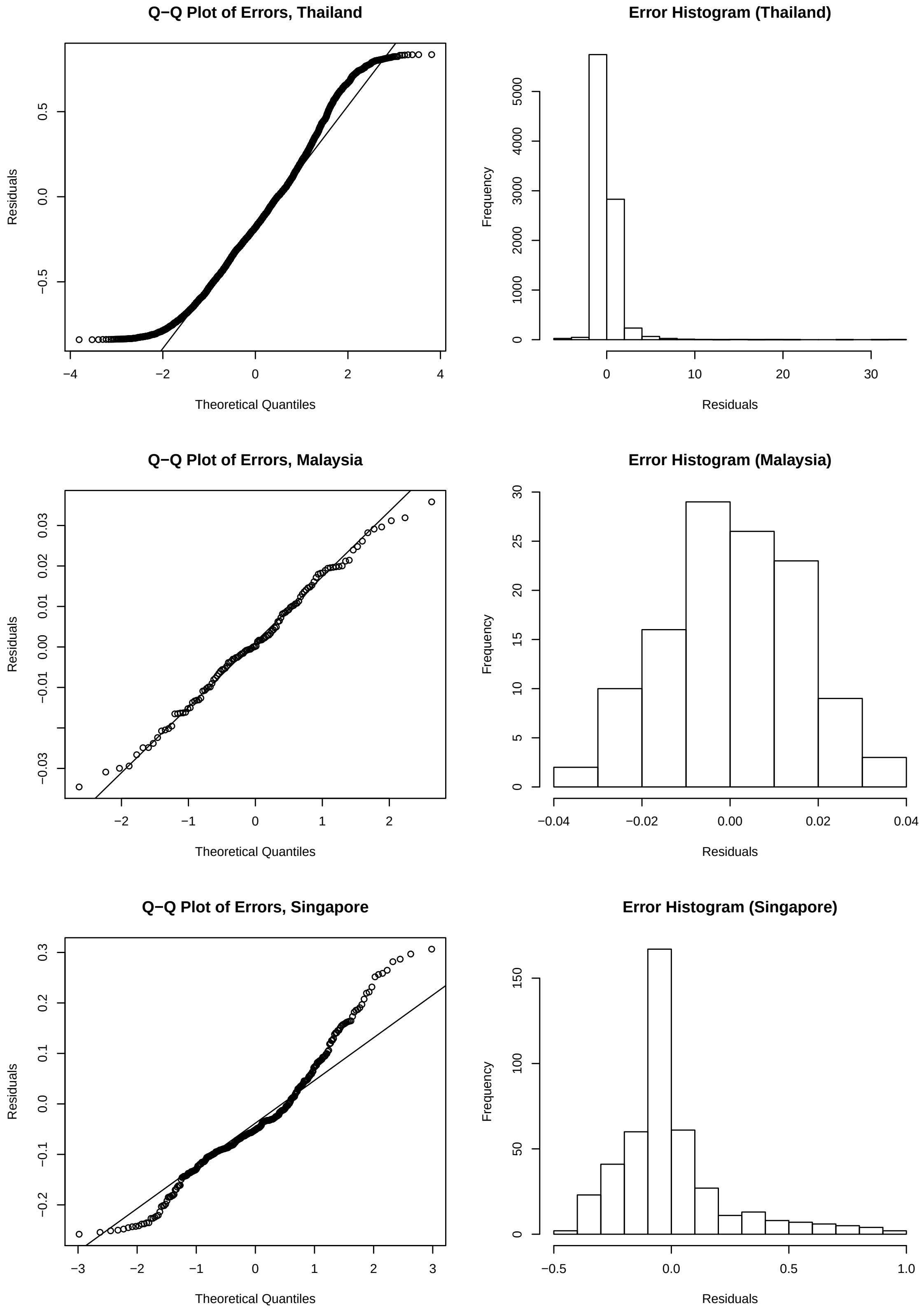

Figure 1
