# Supplementary material for: Impact of sars-cov-2 interventions on dengue transmission
Source: PLoS Negl Trop Dis. 2020 Oct 29;14(10):e0008719. doi: 10.1371/journal.pntd.0008719 (PMC7595279; doi:10.1371/journal.pntd.0008719)
Supplement: S2 Appendix — (DOCX) [file pntd.0008719.s002.docx]

| **Country** | **Social Distancing Measures** | **Implementation Date**  **(as of 18-05-2020)** |
| --- | --- | --- |
| **Malaysia** | *Movement Control Order* | |
|  | - General prohibition of mass movements and gatherings across the country including religious, sports, social and cultural activities. | 18-03-2020 to 04-05-2020  Extended to 12-05-2020 on 23-04-2020 |
|  | - Closure of borders | 18-03-2020 to 04-05-2020  Extended to 14-04-2020 on 25-03-2020 |
|  | - Closure of all schools and institutions | Extended to 28-04-2020 on 10-04-2020 |
|  | - Closure of all government and private premises except those involved in essential services | Extended to 12-05-2020 on 23-04-2020 |
|  | *Enhanced Movement Control Order* |  |
|  | - All residents and visitors within the area are forbidden from exiting their home - Lockdown on specific area - All businesses are shut down; adequate food supplies is given to residents by authorities and medical base is established within the area | 27-03-2020 onwards  (14-day restriction on specific areas if large cluster is detected*)* |
|  | *Conditional Movement Control Order (Relaxed Lockdown)* | |
|  | - All public transport services resume - Two family members will be allowed to buy food and other daily essentials - Reopening of certain businesses | 04-05-2020 to 12-06-2020 |
| **Singapore** | - Closure of certain entertainment venues - Groups must not exceed 10 people - At least 1m physical spacing in public venues (e.g. queuing, eating) - Tuition and enrichment centres, faith-based activities, events are suspended - SHNs for UK/US returnees | 27-03-2020 to 06-04-2020 |
|  | Circuit Breaker Measures   - Only takeaway and delivery are is allowed at all food places - Physical retail outlets offering non-essential services are closed - Closure of schools - Closure of workplaces unless deemed essential services - Closure of all recreational venues and places of worship - Government advises public to wear masks - Public is advised to stay home unless for essential purposes | 07-04-2020 to 21-04-2020 |
|  | Stricter/Extended Circuit Breaker   - Public advised to go out alone - More businesses are closed (e.g. stalls selling mostly beverages/desserts, barbers, hairdressers, TCM/acupuncture) - Additional restrictions placed on businesses (e.g. optician visits by appointment only) - Controlled access at areas susceptible to crowding (e.g markets) - Increased enforcement at work premises | 22-04-2020 to 11-05-2020 |
|  | Extended Circuit Breaker   - Opening of selected retail businesses - Home-based food businesses are allowed to operate | 12-05-2020 to 01-06-2020 |
| **Thailand** | Emergency Decree Invoked   - Closures of various businesses and venues in most parts of the country, a mask requirement on public transportation, and travel restrictions to and from some provinces. - Inclusion of night time curfew of between 10pm and 4am and inbound travel restrictions | 26-03-2020 to 30-04-2020 |
|  | Extension of State of Emergency   - The night time curfew of between 10pm and 4am and inbound travel restrictions will remain in place. - An extension to the ban on all inbound commercial flights except for repatriation, cargo and emergency landings | Extended to 31-05-2020 on 28-04-2020 |
|  | Easing of Lockdown Measures   - Restaurants, hair salons, parks, markets, open-air sports venues such as tennis courts, golf courses, shooting and archery ranges, and pet grooming salons and nurseries are reopening nationwide - Social distancing rules are still in effect and are strictly adhered to. Restaurants need to seat their customers 1.5m apart from one another - At salons and barbershops, waiting inside is not allowed. Each customer must make an appointment, while shops are required to be cleaned for 20 minutes after each one of the two-hour slots. | From 03-05-2020 Onwards |
